# The disappearing San of southeastern Africa and their genetic affinities

**DOI:** 10.1007/s00439-016-1729-8

**Published:** 2016-09-20

**Authors:** Carina M. Schlebusch, Frans Prins, Marlize Lombard, Mattias Jakobsson, Himla Soodyall

**Affiliations:** 1 Department of Organismal Biology, Evolutionary Biology Centre, Uppsala University, Norbyvägen 18C, SE-752 36 Uppsala, Sweden; 2 School of Anthropology, Gender and Historical Studies, University of KwaZulu-Natal, Durban, South Africa; 3 Department of Anthropology and Development Studies, University of Johannesburg, Auckland Park, 2006, Johannesburg, South Africa; 4 Science for Life Laboratory, Uppsala University, Norbyvägen 18C, SE-752 36 Uppsala, Sweden; 5 Division of Human Genetics, School of Pathology, Faculty of Health Sciences, University of the Witwatersrand, and National Health Laboratory Service, Johannesburg, South Africa

## Abstract

Southern Africa was likely exclusively inhabited by San hunter-gatherers before ~2000 years ago. Around that time, East African groups assimilated with local San groups and gave rise to the Khoekhoe herders. Subsequently, Bantu-speaking farmers, arriving from the north (~1800 years ago), assimilated and displaced San and Khoekhoe groups, a process that intensified with the arrival of European colonists ~350 years ago. In contrast to the western parts of southern Africa, where several Khoe-San groups still live today, the eastern parts are largely populated by Bantu speakers and individuals of non-African descent. Only a few scattered groups with oral traditions of Khoe-San ancestry remain. Advances in genetic research open up new ways to understand the population history of southeastern Africa. We investigate the genomic variation of the remaining individuals from two South African groups with oral histories connecting them to eastern San groups, i.e., the San from Lake Chrissie and the Duma San of the uKhahlamba-Drakensberg. Using ~2.2 million genetic markers, combined with comparative published data sets, we show that the Lake Chrissie San have genetic ancestry from both Khoe-San (likely the ||Xegwi San) and Bantu speakers. Specifically, we found that the Lake Chrissie San are closely related to the current southern San groups (i.e., the Karretjie people). Duma San individuals, on the other hand, were genetically similar to southeastern Bantu speakers from South Africa. This study illustrates how genetic tools can be used to assess hypotheses about the ancestry of people who seemingly lost their historic roots, only recalling a vague oral tradition of their origin.

**Electronic supplementary material:**

The online version of this article (doi:10.1007/s00439-016-1729-8) contains supplementary material, which is available to authorized users.

## Introduction

**Fig. 1:**
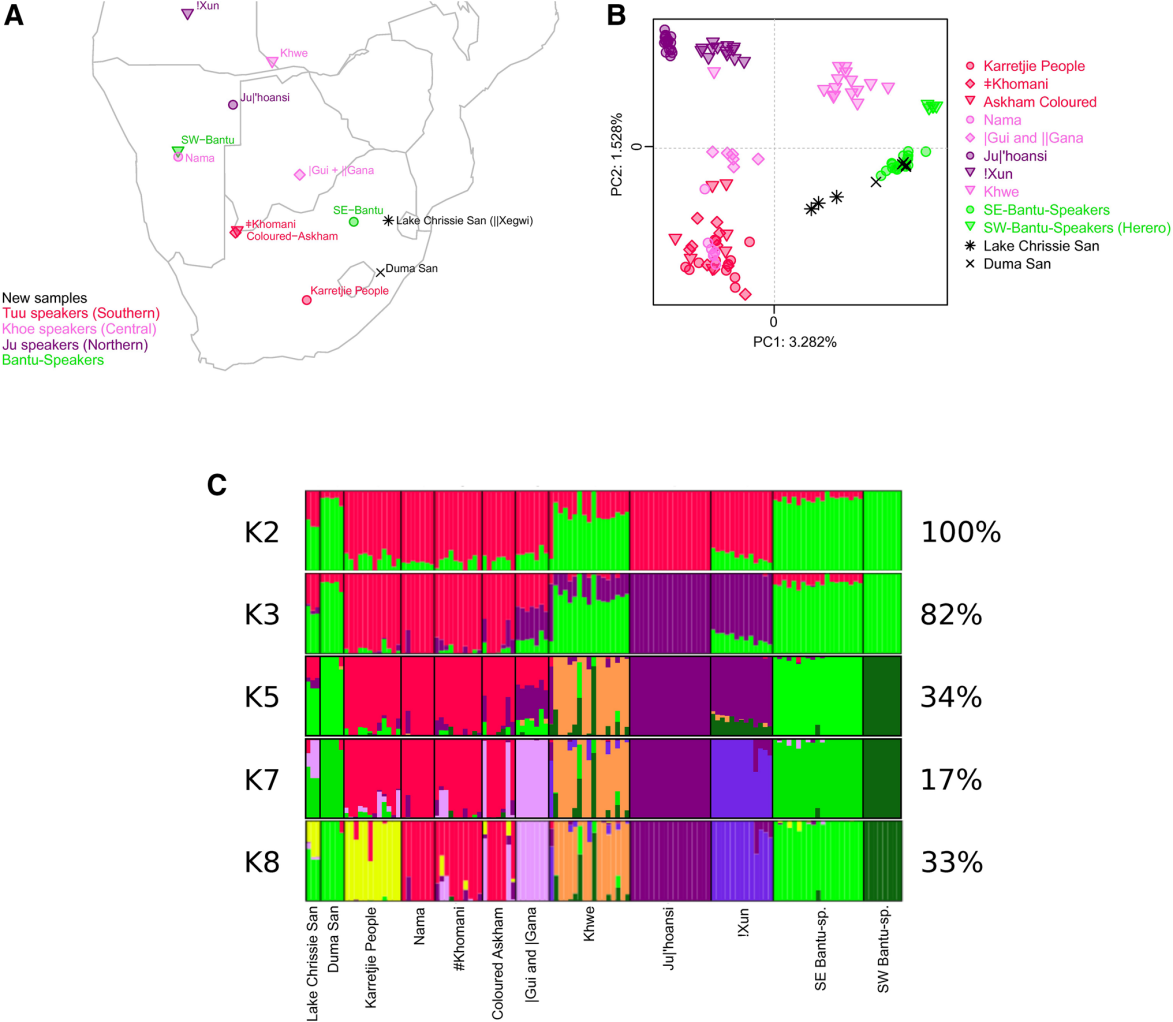
Distribution and population structure of the southern African data set. **a** Geographical locations of new samples (*black*) and comparative data populations from Schlebusch et al. (2012). **b** Principal component (PC) analysis, showing PC1 and PC2. PC1 and PC2 were flipped to correspond to geography. For full PCA (PC1 to PC8) and unmodified PC1 + 2 of the southern African data set, see Fig. S2. **c** Admixture analysis of the southern African data set showing K2, K3, K5, K7, and K8. For full admixture analysis (K2-10), see Fig. S3

The history of the San and Khoekhoe groups (sometimes also referred to as Khoisan, Bushmen, or Batwa—see Online Resource 1, Supplementary Note 1 on the terminology used in the article) in the eastern parts of southern Africa remains poorly understood. There is a continuous loss of oral traditions, and only fragmentary documentation by European settlers arrives a few hundred years ago (Adhikari 2010; Vinnicombe 1976; Wright 1971). Unlike the Kalahari San of the western parts of southern Africa, most of the southeastern groups disappeared before detailed anthropological studies could be undertaken. Thus, the origins and affinities of the groups and individuals with oral traditions of San ancestry, such as the Lake Chrissie San and the Duma San groups of South Africa, are uncertain. In the 1950s, there were only about 50 San individuals left near Lake Chrissie (Fig. 1; Fig. S1) (Barnard 1992; Potgieter 1955; Ziervogel 1955). Most of the older generation still knew their own San language, ||Xegwi, at the time. Today, only a few individuals still recognize their San ancestry, and no one speaks the language or knows the ||Xegwi cultural practices (see Online Resource 1, Supplementary Note 2 for a more comprehensive review of ||Xegwi history). It has been suggested that the ||Xegwi were remnant individuals from the original ‘Transvaal’ San (Sanders 2013; Schoonraad and Schoonraad 1972), such as those who inhabited the Honingklip Shelter in Mpumalanga (Korsman and Plug 1992), scattered refugee groups from the Free State Province (Potgieter 1955; Prins 1999, 2001), and/or groups from the uKhahlamba-Drakensberg of Lesotho (Mitchell 1990; Prins 1999, 2001). These groups fled from the in-coming European settlers and the turmoil that resulted from clashes between settlers and Bantu-speaking farmers. Historical documents record a large group of San individuals migrating from the central uKhahlamba-Drakensberg to the Highveld north of the Vaal River (southern Transvaal Highveld) (Filter 1925; Prins 1999, 2001), and they could represent a large part of the more recent San groups from Lake Chrissie. This inference is corroborated by the fact that the second language spoken by the San of Lake Chrissie was Southern Sotho, which is spoken by people from Lesotho and surrounding areas (Lanham and Hallowes 1956; Potgieter 1955; Prins 1999, 2001).

The ‘Mountain Bushmen’ (Walsham How 1962) or ‘People of the Eland’ (Vinnicombe 1976) were groups of San individuals who inhabited the mountainous regions of the current Lesotho, KwaZulu-Natal, Griqualand East, and the former Transkei (Barnard 1992). Walsham How (1962) reports that when the first Sotho-speaking farmers arrived in Lesotho at about 1600 AD, they found the region (mostly the lowlands) occupied by nomadic San hunter-gatherers. Initially, hunter-gatherers and farmers lived without much conflict, and occasionally, Sotho men would take San women as wives, or employ young San men for herding cattle (Walsham How 1962). As a result of encroaching Bantu-speaking farmers from the north and European settlers from the south-west, the San of southeastern Africa was mostly confined to the high mountains in and around current-day Lesotho (Wright 1971), where the Later Stone Age archaeological record attest to the long-term hunter-gatherer occupation. For example, at Melikane Rock Shelter, there is evidence of hunter-gatherers from at least about 80,000 years ago (Stewart et al. 2012). Here, in the mountains, the San hunted relatively undisturbed until game became scarce, and they began stealing livestock from the Bantu-speaking and European farmers. Both the latter groups retaliated by ‘hunting’ and executing San men, while San women and children were often taken as prisoners and servants, causing those who remained to flee or seek protection (Walsham How 1962; Wright 1971). A San language was still much spoken in Lesotho by the 1870s, and before 1879, there were still numerous San individuals in southern Lesotho. Subsequently, their numbers dwindled as a result of military campaigns amongst Bantu-speaking groups (Sotho and Nguni groups), between Bantu-speaking and European farmers, and as a result of farmers (Bantu-speaking and European) retaliating cattle theft by killing San (Walsham How 1962; Wright 1971). Some San groups, such as the !Gã !ne (in the Eastern Cape section of the Maloti Drakensberg) and the amaThola (who frequented the area between the Lotheni and Mzimvubu rivers) experienced rapid social and genetic transformation. Historical documentation clearly indicates that these last surviving San incorporated non-San as active members of the group resulting in an admixed group (Challis 2008; Prins 2009). By the late 1880s, the forces of colonialism finally led to the “disappearance” of independent San groups in the Maloti Drakensberg region. Those groups who were not obliterated by violent conflict, either absconded the region altogether or were assimilated by their Bantu-speaking neighbors. Although individuals survived for a while, by the twentieth century, the San of the region was thought to have disappeared (Wright 1971). The Duma San are thought to be remnants of the amaThola and other admixed San groups now residing in KwaZulu-Natal, South Africa (see Online Resource 1, Supplementary Note 3 for a more comprehensive review of Duma San history).

Today, skeletal material from archaeological contexts interpreted as a mixture between San and African farmers (Beaumont 1967; Galloway 1936; Murray 1933; Wells and Dart 1934), and residual Khoe-San physical and linguistic features amongst many southeastern Bantu-speaking groups (Bourquin 1951; De Villiers 1968; Greenberg 1966) are considered as bearing testimony to admixture between the hunter-gathers and farmers (Vinnicombe 1976). Indeed, the previous genetic work attests to such admixture (Pickrell et al. 2012; Schlebusch et al. 2012). In addition, the recent work has shown that San hunter-gatherers of southern Africa also admixed with East African pastoralists, before they came into contact with Bantu-speaking farmers (Breton et al. 2014; Macholdt et al. 2014a; Schlebusch et al. 2012), probably resulting in what is today known as the Khoekhoe population of southern Africa, who are traditionally associated with a herding lifeway, but who still hunted and gathered wild plant foods. Admixture, socio-economic diffusion of hunting and herding lifeways, linguistic affinities, and shared worldviews between the various San and Khoekhoe groups of southern Africa resulted in the currently used collective term ‘Khoe-San’. Distinguishing between populations, for example, to determine specific group relations or closest affinities, is becoming increasingly difficult as a result of lost traditions and merged cultural and genetic histories.

Linguistic classification is the traditional method used to help decipher Khoe-San population histories and relationships (Fig. S1; Table S1) (Güldemann 2014). The Khoisan languages of southern Africa can be seen as a Sprachbund^1^ represented by three independent language families; northern Khoisan (Ju family:^2^ northeastern Botswana, northern Namibia, southern Angola); southern Khoisan (Tuu family: southern Botswana and South Africa); and an additional linguistic family (Khoe-Kwadi) spoken by San groups in Botswana and the KhoeKhoe speaking herders of Namibia and South Africa (such as the Nama) (Fig. S1; Table S1) (Güldemann 2014). Today, remnant Khoe-San groups still live in a wide geographic region across southwestern Africa, extending from southern Angola in the north to the Western Cape Province (South Africa) in the south. The Khoe-San peoples of southeastern Africa have to a large extent lost their identities, and have integrated into other populations.

Most of the San hunter-gatherer groups who used to occupy the geographic region, now known as South Africa, belonged to the !Ui branch of the Tuu family (southern Khoisan) (Table S1; Fig. S1; Fig. 1). In historical times, !Ui dialect clusters were spoken throughout all the parts of the interior of South Africa. The geographic range of these languages stretched from Namaqualand in the west through the Northern Cape Province, the Free State Province and Lesotho, to KwaZulu-Natal and the southeastern parts of Mpumalanga Province in the east (Fig. S1). In the west, the N||ng language still has a few active speakers amongst the ǂKhomani people; however, the ǂKhomani group represent a recently forged community, whose members have a complex and diverse origin and ancestry (see Online Resource 1, Supplementary Note 5 for additional notes on the ǂKhomani). Although an extinct language, the best documented !Ui language is |Xam, a language mainly spoken in the Karoo region, south of the Orange River. The Karretjie people, itinerant groups who today live in the Cape Karoo, are believed to be decedents of |Xam speakers (see Online Resource 1, Supplementary Note 4 and (Schlebusch et al. 2011) for additional notes on the Karretjie People). There were, however, other !Ui dialect clusters in South Africa (Fig. S1; Table S1). A few of the languages from these dialect clusters were recorded, and still had some active speakers in the recent history, such as ||Xegwi from Lake Chrissie. Of the other !Ui dialects and dialect clusters, very little, other than a name, is known, for example, ǂUngkue of the Warrenton-Windsorton area in the northern Cape and !Gã!ne of the eastern Cape region (Traill 1996).

Recent genetic studies on African and global population structure constantly identified Khoe-San groups as the most divergent group of modern humans (split dated to ≥100 kya, or ≥200 using the pedigree-based mutation rate) (Gronau et al. 2011; Schlebusch et al. 2012; Veeramah et al. 2011). Furthermore, among Khoe-San groups, the level of stratification suggests deep separation between northern (Ju speakers) and southern (Tuu speakers) Khoe-San, dating to ~35 kya (Schlebusch et al. 2012). It is thus important to trace the genetic affinities of disappearing Khoe-San groups, such as the Lake Chrissie San and the Duma San. Such work contributes towards the effort to better understand the genetic diversity of southern African Khoe-San groups, and the history of the people that lived in southern Africa prior to the arrival of European settlers in the 1600s and Bantu-speaking farmers several hundred years earlier. Here, we report, for the first time, on the genetic affinities of a few remaining individuals from two southeastern groups with remnant San oral traditions, namely, the Lake Chrissie San and the Duma San of the uKhahlamba-Drakensberg.

## Results

We genotyped ~2.2 million SNPs in three individuals from Lake Chrissie (Chrissie San from here onwards) and five Duma San individuals from the KwaZulu-Natal uKhahlamba-Drakensberg (Duma San from here onwards) (Fig. 1a; “Methods”). The genotype data were combined and analyzed with three different comparative data sets, varying in the number of populations and individuals and the number of overlapping SNPs (Methods and Table S1). The ‘southern African data set’ consisted of the eight new individuals combined with data from Schlebusch et al. (2012), which added an additional 117 southern Africa individuals from eight Khoe-San and two Bantu-speaking populations (Fig. 1a). The ‘KGP extended data set further added two groups of West African ancestry, one East African, and three non-African populations from the 1000 genomes Omni2.5 M data (Methods and Table S1). Both these data sets contain ~2.1 million SNPs. The third comparative data set, the “East African extended data set”, contained six additional East African populations from Pagani et al. (2012) (“Methods”; Table S1), but contains fewer SNPs (~620,000 SNPs).

To summarize the genetic variation and affinities among the individuals, we used the principal component analysis (PCA) for the southern African data set (Fig. 1b; Fig. S2). The first principal component (PC1) that captures the greatest amount of variation (explaining 3.282 % of the variation) separates Khoe-San individuals from Bantu speakers, and PC2 (1.528 %) separates northern San from southern San individuals. On PC1, the Chrissie San individuals are located about half-way in-between the two extremes. We further used an unsupervised clustering approach (Alexander et al. 2009) to estimate admixture fractions among individuals and to obtain a more detailed picture of the relationships among the individuals. The result from the PCA is also reflected in the admixture analysis assuming two clusters (K2), where the Chrissie San had an average of 41.6 % Khoe-San and 58.4 % Bantu-speaker ancestry (Fig. 1c; Fig. S3). With the inclusion of a non-admixed west African population (YRI-Yoruba) as reference in the KGP-extended data set (Fig. S4), the West African origin Bantu-speaker ancestry in the Chrissie San decreases to 51.3 %, and the Khoe-San ancestry increases to 48.7 % (at K3 in Fig. S4). This difference is because of the cryptic Khoe-San admixture in the southeastern Bantu speakers (SE-Bantu in the figure) and southwestern Bantu speakers (SW-Bantu in the figure) that becomes visible with the inclusion of a non-admixed West African group.

The Duma San individuals on the other hand clearly grouped with southeastern Bantu speakers in both the PC and the admixture analysis. Although Khoe-San admixture among the Duma San individuals is visible (mean of 11.33 % at K2 in Fig. 1c and Fig. S3 and mean of 19.35 % at K3, Fig. S4), it was not significantly greater (*p* values >0.722, Mann–Whitney *U* test) than the level of Khoe-San admixture in the southeastern Bantu-speaking populations (mean of 9.03 % at K2 in Fig. 1c and Fig. S3 and mean of 18.97 % at K3, Fig. S4). This observation of similar amounts of Khoe-San admixture in Duma San compared to southeastern Bantu speakers is also confirmed by the admixture analysis at the level, where southeastern Bantu speakers form their own cluster (K8, Fig. S4, and K5, Fig. 1c and Fig. S3—light green cluster). At this level of clustering, most Duma San ancestry are assigned entirely to the southeastern Bantu-speaker cluster (light green cluster), whereas a large part of the Chrissie San ancestry can be assigned to a Khoe-San cluster (red cluster).

The Khoe-San ancestry fraction of the Chrissie San individuals clearly group with southern San individuals (red cluster from K3 and onwards—Fig. 1c; Fig. S3) and not with northern San (dark purple cluster from K3 and onwards). This is also visible on PC2 of the PCA (Fig. 1b; Fig. S2). Finer level clustering seems to indicate an affiliation of the Chrissie San with the Karretjie people (K8—Fig. 1c and Fig. S3, and PC7—Fig. S2), rather than with the other groups who show southern San ancestry (e.g., ǂKhomani, Askham Coloured, or Nama). However, PC6 (Fig. S2) also show some affinity between the Chrissie San and the Kalahari Khoe-Speaking central San groups |Gui and ||Gana. This association of the Chrissie San with the |Gui and ||Gana is also seen at K7 (Fig. 1c). The Karretjie individuals have not been assigned into a separate cluster at the K7 level, and the Chrissie San are grouping here with the |Gui and ||Gana, rather than with the red cluster that collects the majority of the ancestry of Karretjie people, ǂKhomani, and Askham Coloured and Nama individuals. Assuming eight clusters, the Karretjie people individuals receive their own cluster (K8 in Fig. 1c). At this level of resolution, the Chrissie San groups with the Karretjie people rather than the |Gui and ||Gana, suggesting stronger affinity to the Karretjie people than to the |Gui and ||Gana.

The introduction of pastoralist practices and the appearance of Khoekhoe groups in southern Africa ~2000 years ago have been associated with a migration of people from East Africa followed by admixture with indigenous San groups (Breton et al. 2014; Macholdt et al. 2014a, b; Schlebusch et al. 2012). For instance, the Nama (a Khoekhoe group) have a distinct fraction, 10–15 % of their ancestry tracing to the East African migrants. Both the Chrissie San and the Duma San do not show this pattern of a limited east African ancestry (neither in the PC analysis nor the admixture analysis), while this component was clearly visible in the Nama (blue component—K5—Fig. S4) and to some extent the ǂKhomani and Coloured-Askham populations. An f3 test for east African admixture in the Chrissie San also confirmed the absence of East African ancestry (positive *Z* scores, Table S3a). This absence of the East African component in the Chrissie San is further confirmed by the admixture analysis of the extended east African data set, which contains six more East African comparative groups (Fig. S5). The East African component in the Nama is seen here to associate the strongest with the Amhara and Oromo populations from Ethiopia (K11-12, Fig. S5). From the admixture analysis, no East African component was found in the Duma San; however, an f3 test for East African ancestry indicated some level of admixture (negative *Z* scores, Table S3a). Since the Duma San appear very similar to southeastern Bantu speakers in the admixture analysis, we also tested the southeastern Bantu speakers for East African ancestry (Table S3a, S3b) and found similar signals of East African admixture (Table S3b). Thus, the East African admixture observed in the Duma San samples is likely of the same origin as the East African admixture in the southeastern Bantu speakers. The Duma San further show weak evidence of non-African admixture (slightly negative *Z* scores, Table S3a) that is not observed in Bantu speakers (Table S3a, S3b).

The West African ancestry fraction of Chrissie San and Duma San is clearly more associated with southeastern Bantu speakers from South Africa compared to southwestern Bantu speakers from Namibia (Fig. 1c; Fig. S3). An f3 test with (presumably) non-admixed Khoe-San (Ju|’hoansi) individuals and West African Yoruba individuals as source populations indicates admixture between West African groups (Bantu speakers) and Khoe-San in both Duma San and Chrissie San (negative *Z* scores in both Duma San and Chrissie San, Table S3a, S3b). We also dated the Khoe-San and Bantu-speaker admixture events in the Duma San (Table S4), using admixture linkage disequilibrium decay curves, and obtained a date of 33 generations (0.002885 residual SE) which translates to 830 years ago (25 years/generation) for Duma San (Table S4). This admixture date is very similar to the southeastern Bantu-speaker admixture date (31 generations), but different from other Khoe-San and southwestern Bantu-speaker groups’ admixture dates (Table S4). The Chrissie San admixture date could not be determined due to a too small sample size.

## Discussion

The three Chrissie San individuals, who had oral and recorded histories of being descendants of the ||Xegwi from Lake Chrissie (see Online Resource 1, Supplementary note 2 on the ||Xegwi), clearly had a distinct and substantial Khoe-San genetic component. All the three Chrissie San individuals show close to half of their ancestry tracing to Bantu-speaking groups and half of their ancestry tracing to Khoe-San groups, which could be placed into context of other Khoe-San groups by comparison with published genetic data. The Khoe-San component of the Chrissie San showed a clear affinity to the descendants of southern San groups (e.g., ǂKhomani and Karretjie people). Within the southern San cluster, the Chrissie San genomes showed similarity to the Karretjie people (rather than the ǂKhomani), and also to the |Gui and ||Gana groups from Botswana. This affinity is not unexpected, considering that these two groups are the geographically closest groups in the comparative data to the historical known area of the ||Xegwi (Karretjie people—702 km and |Gui + ||Gana—631 km, vs. the ǂKhomani—939 km). Furthermore, our findings align with linguistic inferences, since the ||Xegwi language belongs to the same linguistic branch (!Ui of the Tuu family) as the language of the probable ancestors of the Karretjie people (|Xam). The evidence of admixture from southeastern Bantu speakers was not surprising, since some of the investigated individuals reported Swazi (Bantu speaker) parents or grandparents and the current-day Chrissie San speak the Swazi language (although their historic language were ||Xegwi and Southern Sotho—see Online Resource 1, Supplementary note 2). The combined Southern San and Bantu-speaker ancestry of the Lake Chrissie San is also apparent in the affinities of their mtDNA and Y-chromosome haplogroups (see previously published results in (Schlebusch et al. 2013) and (Naidoo et al. 2010) and Online Resource 1, Supplementary Note 6). While all the three Lake Chrissie individuals carry mtDNA haplogroups likely autochthonous to the region (L0d and L0a haplogroups), their Y-chromosome haplogroups (E1b1a1 and B2a) suggest paternal line contributions from Bantu speakers.

Aside from Southern San and Bantu-speaking ancestry, we did not find other significant ancestry contributions (i.e., eastern African or European) in the Chrissie San. It has been shown previously that the genetic makeup of the Nama (a Khoekhoe herding group) is best explained by admixture between a southern San group and a group that migrated from East Africa that brought herding practices to southern Africa (Breton et al. 2014; Macholdt et al. 2014a). There is also a linguistic hypothesis that the Khoe-Kwadi language family emerged through contact between southern African languages and an immigrant language group, possibly associated with the introduction of herding to southern Africa (Güldemann 2008). From historical records, it is known that Khoekhoe herding groups, such as the Nama, Eini, !Ora, and Cape Khoekhoe groups, occupied the western parts of South Africa (Fig. S1) (Barnard 1992), although it is still unclear if the Khoekhoe range extended into the more central and eastern parts of southern Africa (north of the Orange river and perhaps the Vaal river) (Ehret 2008). Chrissie San individuals do not harbor genetic material from East African groups, in contrast to, for example, the Nama. This result does not rule out that Khoekhoe people were present in these areas, since the ||Xegwi were hunter-gathering San and not Khoekhoe herders. The absence of East African admixture, however, indicates that: (1) there were no Khoekhoe groups for long periods in this region or (2) the ||Xegwi San lived separated from potential Khoekhoe groups in the region.

In contrast to the Chrissie San, the Duma San individuals showed a genetic background very similar to southeastern Bantu speakers—with low levels of Khoe-San admixture similar to the levels in southeastern Bantu speakers. The admixture into Duma San was dated to 830 years ago and is consistent with southeastern Bantu speakers arriving in the interior parts of the KwaZulu-Natal region of South Africa during the early stages of the second millennium AD (Ribot et al. 2010). It, therefore, seems that the oral history of San ancestry in this group was not clearly distinguishable in their genetic ancestry. Nonetheless, the Duma San do contain a fraction of San ancestry (even though it was at similar levels as southeastern Bantu speakers) and it might be that the Duma San kept an oral tradition of their San ancestry, while in the rest of the southeastern Bantu speakers, this history was lost. Duma San Y-chromosomes belonged to the Bantu speaker associated haplogroups, E1b1a1 and B2a, and their mtDNA haplogroups were from both Bantu speaker (L3 and L2a) and Khoe-San (L0d) origin (see (Schlebusch et al. 2013) and (Naidoo et al. 2010) and Online Resource 1, Supplementary Note 6). We, furthermore, detected the evidence of East African admixture in the Duma San. This East African ancestry, however, was also found in the southeastern Bantu speakers, and it is likely a consequence of their migration route, where southeastern Bantu speakers have been following a route through East Africa during the “Bantu expansion”, starting from West Africa (Guthrie 1948; Holden 2002; Li et al. 2014). Furthermore, historical documentation also shows that the amaThola (who later became the Duma San) incorporated Cape Khoekhoe into their group (Challis 2008). The East African ancestry in the Duma San could, therefore, stem from either or both of these sources (i.e., southeastern Bantu speakers and Cape Khoekhoe). Furthermore, the Duma San show weak evidence of non-African admixture that is not observed in Bantu speakers. This weak signal of non-African admixture possibly points to the oral histories of Indian and European admixture in the Duma San (also see Online Resource 1, Supplementary note 3 on the complex history and diverse origins of the Duma San).

This study illustrates how genetic tools can be used to pinpoint the genetic ancestry of people who lost their historic roots and who only recalled a vague oral tradition of their ancestry. In certain cases, oral traditions and genetic ancestry do not correlate, as was seen in the case of the Duma San, but in the case of the Lake Chrissie ||Xegwi descendants (Chrissie San), we could clearly distinguish Khoe-San ancestry. We illustrated that the ||Xegwi of Lake Chrissie were genetically related to southern San groups, which is in agreement with written records that the ||Xegwi spoke a language that grouped into the !Ui sub-group of Tuu (southern Khoisan) (Fig. S1; Table S1). Among southern San groups, the ||Xegwi are closer affiliated genetically with the descendants of |Xam speakers (Karretjie people), compared to the descendants of N||ng speakers (ǂKhomani), but the ||Xegwi of Lake Chrissie also showed genetic affinity to Botswana San groups. Thus, although most of the southern San groups of South Africa (who spoke Tuu languages) are culturally extinct today, we can add their genetic variation to the known range of human genetic variation by including their descendant groups in the genetic analysis. This was previously done for the ǂKhomani and the Karretjie people, who were demonstrated to be representatives of southern San groups, and who separated around 35 kya from northern San groups (e.g., the Ju|’hoansi from Namibia). This study adds to the diversity of human genetic variation across the world and reveals the population history of the lost southeastern San groups, such as the descendants of the ||Xegwi from Lake Chrissie.

## Methods

### Sampling and genotyping

DNA samples used in this study have been collected during two field trips to Kamberg (in the KwaZulu-Natal Province of South Africa) and Lake Chrissie (in the Mpumalanga Province of South Africa) (Fig. 1a). The project was approved by the Human Research Ethics Committee (Medical) at the University of the Witwatersrand, Johannesburg (Protocol Number: M050902). Informed consent was obtained from all individual participants included in the study. Peripheral blood was collected in EDTA tubes. DNA was extracted using a salting-out method (Miller et al. 1988). Three unrelated individuals with an oral history of ||Xegwi ancestry from Lake Chrissie and five unrelated individuals with an oral history of Duma San ancestry were selected for SNP typing. Samples were genotyped on the Illumina Omni2.5 M (HumanOmni25-8v1-2_A1) SNP chip. Genotyping was performed by the SNP&SEQ Technology Platform in Uppsala, Sweden (www.genotyping.se). Results were analyzed using the software GenomeStudio 2011.1, and the data were exported to Plink format and aligned to Human Genome build version 37. Genotype data are available from the ArrayExpress database (http://www.ebi.ac.uk/arrayexpress) and on the research group homepage (http://jakobssonlab.iob.uu.se/data/).

### SNP data processing and filtering

SNP data quality filtering and merging to comparative data was done with PLINK v1.90b3 (Chang et al. 2015). A 10 % genotype missingness threshold was applied, and the HWE rejection confidence level was set to 0.001. SNPs with a chromosome position of 0, indels, duplicate-, mitochondrial-, and sex chromosome SNPs were removed. All individuals passed a missingness threshold of 15 % and a pairwise IBS threshold of 0.25 (for identification of potential relatives). The resultant data set of 2257,682 SNPs and eight individuals was merged with data from Schlebusch et al. 2012 (Schlebusch et al. 2012), containing 2286,795 quality-filtered autosomal SNPs typed in 117 southern African Khoe-San and Bantu speakers. Before merging the data sets, AT and CG SNPs were removed from the data sets. During the merge the strands of mismatching, SNPs were flipped once, the remaining mismatches were removed, and only the intersection between the data sets were kept. The resultant “southern African data set” contained 125 individuals form 12 populations and 2109,357 SNPs.

To get a more extensive set of African and non-African comparative data, we, furthermore, downloaded SNP data (typed on a similar Illumina Omni chip as our data), from the 1000 Genomes Project website, at ftp://ftp.1000genomes.ebi.ac.uk/vol1/ftp/technical/working/20120131_omni_genotypes_and_intensities/ (Auton et al. 2015). The 1000 genomes genotype data were quality filtered using the same thresholds as used in our data sets (described above). The following populations were included from the 1000 genomes data set: YRI and LWK (Yoruba and Luhya—West African ancestry), MKK (Maasai—East African), and TSI, CEU, and JPT (Tuscans, northeast European ancestry, Japanese—non-African). All populations were randomly down-sampled to 20 individuals. This merged “KGP extended data set” included a total of 2104,593 high-quality SNPs in 245 individuals from 18 populations.

To include additional East African comparative data, the KGP extended data set was then merged with data from Pagani et al., (Pagani et al. 2012). Since the Pagani data were mapped to hg18, we converted the positions to hg19 to match the previous data sets, using the *LiftOver* tool (https://genome.ucsc.edu/cgi-bin/hgLiftOver). The Pagani et al. data were quality filtered and merged to the combined data set in the same way as described above. The following populations were included from the Pagani data set: Amhara (Ethiopia, Semitic), Ari-Blacksmith (Ethiopia, Omotic), Gumuz (Ethiopia, Nilotic), Oromo (Ethiopia, Cushitic), Somali (Somalia), and Sudanese (South Sudan). All populations from Pagani et al., containing more than 20 individuals, were randomly down-sampled to 20 individuals. Since the Pagani et al. data set was generated on an Illumina Omni 1 M chip, less SNPs remained after merging with our data sets. This merged “East African extended data set”, therefore, contained 627,777 variants in 354 individuals from 24 populations.

### Population genetic analysis

The population genetic analysis was conducted for all the three different data sets: (1) the southern African data set (8 new individuals combined with Schlebusch et al. data), containing 125 individuals form 12 populations and 2109,357 SNPs; (2) the KGP extended data set (southern African data set combined with 1000 genomes Omni2.5 M data), containing 245 individuals from 18 populations and 2104,593 SNPs; and (3) East African extended data set (KGP extended data set combined with Pagani et al. data), containing 354 individuals form 24 populations and 627,777 SNPs.

We inferred admixture fractions (Alexander et al. 2009) to investigate genomic relationships among individuals based on the SNP genotypes. Default settings and a random seed were used. Between 2 and 15 clusters (*K*) were tested (*K* = 2 to 15). A total of 100 iterations of ADMIXTURE were run for each value of K, and the iterations were analyzed using CLUMPP (Jakobsson and Rosenberg 2007) for each K to identify common modes among replicates; the LargeKGreedy algorithm with 1000 repeats was used. Pairs of replicates yielding a symmetric coefficient *G′* ≥ 0.9 were considered to belong to common modes. The most frequent common modes were selected, and CLUMPP was run a second time for all values of K containing the most frequent common mode (LargeKGreedy algorithm, 10,000 repeats). The results were visualized using DISTRUCT (Rosenberg 2004).

PCA was performed with EIGENSOFT (Patterson et al. 2006; Price et al. 2006) with the following parameters: *r*2 threshold of 0.2, population size limit of 20, and 10 iterations of outlier removal.

We estimated the admixture time based on linkage disequilibrium (LD) decay due to admixture (Patterson et al. 2012) and computed additional tests of admixture (f3 tests) (Patterson et al. 2012). Default parameters were used. Various f3 tests were conducted, and various LD decay curves were estimated using different populations as the two parental reference populations (Tables S3, S4). Ju|’hoansi was used as a Khoe-San source population and Yoruba (YRI) as a West African source population to minimize the effect of admixture in the source populations. The standard error was estimated with a jackknife procedure. Generations were converted to years using 25 years per generation.

## Electronic supplementary material

Below is the link to the electronic supplementary material.

Supplementary material 1 (PDF 1120 kb)

## Footnotes

1 The Khoisan languages of southern Africa can be classified as a Sprachbund, i.e., a group of languages that share common areal features but not necessarily separates from a common source.

2 The newer division name of ‘northern Khoisan’ is K’xa (see Online Resource 1, Table S1). The K’xa division includes the Ju division and, in addition, a single, related language, ǂHõa. For the purposes of this article Ju is used in reference to northern Khoisan, since no ǂHõa speakers are present in the dataset.
