# Supplementary material for: The disappearing San of southeastern Africa and their genetic affinities
Source: Hum Genet. 2016 Sep 20;135(12):1365–73. doi: 10.1007/s00439-016-1729-8 (PMC5065584; doi:10.1007/s00439-016-1729-8)
Supplement: Supplementary file 1 — Supplementary material 1 (PDF 1120 kb) [file 439_2016_1729_MOESM1_ESM.pdf]

SUPPLEMENTARY MATERIAL

The disappearing San of southeastern Africa and their genetic affinities

Carina M. Schlebusch[1#], Frans Prins[2], Marlize Lombard[3], Mattias Jakobsson[1,4] and Himla Soodyall[5#]

[1] Department of Evolutionary Biology, Evolutionary Biology Centre, Uppsala University, Norbyvägen 18D, SE-752 36 Uppsala, Sweden

[2] University of KwaZulu-Natal, School of Anthropology, Gender and Historical Studies, Durban, South Africa

[3] Department of Anthropology and Development Studies, University of Johannesburg, Auckland Park, 2006, Johannesburg, South Africa

[4] Science for Life Laboratory, Uppsala University, Norbyvägen 18C, SE-752 36 Uppsala, Sweden

[5] Division of Human Genetics, School of Pathology, Faculty of Health Sciences, University of the Witwatersrand, and National Health Laboratory Service, Johannesburg, South Africa

SUPPLEMENTARY NOTES

Supplementary Note 1

Terminology used in the article

The term ‘Khoisan’ was first used as a biological label by Leonard Schultze (Schultze 1928), and was subsequently popularized by Isaac Schapera (Schapera 1930). Today, it is used to collectively refer to the Khoikhoi (old Nama word) or Khoekhoe (modern Nama word), who used to herd livestock, and the San, who were hunter-gatherers. The word ‘Khoi’ or ‘Khoe’ means ‘person’ in Nama. Two surviving pastoralist groups, the Nama and Korana, use the word ‘Khoenkhoen’, meaning ‘people of the people’. The word ‘San’ is the Khoe word for ‘foragers’ or ‘bushmen’ (Barnard 1992).

In 2002, at a meeting attended by the Working Group of Indigenous Minorities in Southern Africa (WIMSA) and the South African San Council (SASC), the San people decided that they wanted to be referred to by their individual community names (!Xun, ǂKhomani, etc.) or collectively as San. When collectively referring to the San and the Khoe, the term Khoe-San was suggested (Crawhall 2006).

In this article we therefore use ‘San’ when referring collectively to the traditional hunter-gatherers of southern Africa and ‘Khoekhoe’ when referring to traditional pastoralist groups, and we use ‘Khoe-San’ when referring to both populations collectively. When referring to the linguistic grouping, we use ‘Khoisan-speaking’, and when referring to the sub-groupings of the three Khoisan language families, the nomenclature suggested by Güldemann (Güldemann 2014) is followed.

Supplementary Note 2 (drafted by Frans Prins)

The ||Xegwi

Although anthropologists have been aware of the existence of the ||Xegwi on the southern Mpumalanga Highveld for many years (Schapera 1930), they are one of the least known of all Khoe-San groups (Barnard 1992). There are about 30 people left in the greater Lake Chrissie area of the Mpumalanga Province of South Africa, who still refer to themselves as *Boesman* (the old Afrikaans term for the San) or amaBushmana. Most of them are farm laborers, or live in small townships adjacent to the towns of Middelburg, Carolina and Lake Chrissie.

The main source of information on this group is a short monograph by E. F. Potgieter (1955), who recorded around 50 individuals, whom he thought may be of San hunter-gatherer descent (Potgieter 1955). Although often portrayed in popular literature as the last Transvaal San (Sanders 2013; Schoonraad and Schoonraad 1972), oral history, written historical sources and linguistic evidence indicate that the ||Xegwi originally hailed from the Maloti Drakensberg region (Prins 1999, 2001). In fact, it was rather perplexing to Potgieter that some of the ||Xegwi informants, whom he interviewed in the 1950's, spoke Southern Sotho. Thus he suggested a Lesotho or eastern Free State origin for them. Potgieter also documented the Sotho name for the ||Xegwi, which is Tlountle, meaning pretty elephant. Interestingly, a rock shelter with the same local name that contains Later Stone Age deposits is located in western Lesotho. As speculated by Mitchell (1990), the archaeologist who excavated this particular shelter, it is tempting to suggest that this area may have been the original homeland of the ||Xegwi (Mitchell 1990).

||Xegwi descendants interviewed by F. Prins in the late 1990's (1998/1999 field notes) maintain that they originally came from a mountainous area next to the land of the Basotho people. It is said that this land contained large herds of grazing animals but that these were soon depleted by colonial hunters. The San seem to have left the area after they were pursued by local commandos for stealing cattle and horses from European settler farms. Interestingly, these oral traditions are corroborated by the memoirs of Pastor Filter, a German missionary attached to the Luneburg Mission Station, who did regular transport riding between the colony of Natal and the goldfields at Baberton. In the early 1880s he came across a party of San near the present town of Piet Retief who were travelling towards Lake Chrissie. They told him that they were fleeing from angry Boers

after they had stolen livestock from farms in the greater Weenen area at the beginning of the Anglo-Zulu War of 1879. According to Filter, this party consisted of two groups, namely a group who were shorter and more ‘yellow’ in complexion, than a second group who were taller and ‘darker’ in complexion. According to these San, the group who was smaller and lighter originated from Lesotho, whilst the other group originally lived in the foothills of the Central Drakensberg (Filter 1925). Potgieter noticed a similar distinction in stature and hue when he worked amongst the descendants of these San in the Lake Chrissie area some seventy years later (Potgieter 1955).

Some forty years after Potgieter worked amongst the ||Xegwi, they were again visited by F. Prins who noticed that the same physical distinctions still apply. He also found that these distinctions were self-imposed rather than enforced from the outside (F. Prins fieldnotes 1998). Potgieter maintained that the group of taller, darker in complexion, San was the offspring of hunter-gatherers with their Swazi neighbors. He also researched the genealogies of known San families at Lake Chrissie to distinguish those with less admixture from their ‘mixed-descent’ relatives. Potgieter’s (1955) monograph essentially deals with the smaller, lighter in complexion group, whom he considered to be the least admixed San. Informants, however, told Prins that the group who is considered taller and darker in complexion originated earlier than those with a lighter complexion, and that they were already established as a distinct group, before the San left the Maloti-Drakensberg region. According to them, there was intermarriage with the Sotho and other Bantu-speaking (non-Swazi) people long before they migrated to Lake Chrissie. It is also noteworthy that some Swazi groups such as the amaNgqamane historically raided the Lake Chrissie area in search of booty (Prins 1999). This included the children of neighboring tribes including San individuals who were abducted to become serfs known as the *titfunjwa*. These captives formed an important part of the Swazi economy and were assimilated into Swazi society (ibid). Although Swazi men often took San concubines, children of these unions frequently escaped and reunited with independent San groups (F. Prins fieldnotes 1998). By these processes, there was gene flow in both directions.

Sanders noted that the taller and darker in complexion San group of Lake Chrissie often took on the family name of Ngwenya (Sanders 2013). According to him, their San ancestors were most probably acculturated by Sotho families with the name of Kwena who were once settled west of

Mahlangatja in modern-day Swaziland. Whereas admixture with Swazi/Sotho communities in or near Swaziland is not disputed, it is nevertheless meaningful that the name of Kwena (i.e., crocodile) is also a popular totem name adopted by San descendants in Lesotho (F. Prins fieldnotes 2004). In fact, the Mountain San often took on the family name of their African patrons as a survival mechanism in this part of the world (Prins 2009), and it is not surprising that many San descendants have adopted the totem name of the royal Sotho family in Lesotho. Contrary to Sanders's observations, Prins located ||Xegwi descendants with the family name of Ngwenya who regarded themselves as San with little or no Bantu-speaker admixture in 1999.

Today, the original designations according to lighter or darker skin colors of the Lake Chrissie San is largely subjective, and most probably based on individual perceptions and self-designated assignments rather than genealogical criteria as was so meticulously recorded and argued for by Potgieter (1955). Oral data certainly support the notion of intermarriage and subsequent admixture going back many generations, and the "Last Bushmen of Lake Chrissie" may have more diverse genetic origins than was originally postulated. Nevertheless, present notions of a San identity, amongst the now fast-disappearing ||Xegwi, is based on a history of common origin in the mountains adjacent to the land of the Sotho, some physical but varied attributes, and a self-ascribed sense of 'otherness'.

Supplementary Note 3 (drafted by Frans Prins)

The Duma San

The Duma is the largest group of people in the Drakensberg region, who still have some form of self-ascribed San identity. Today, they are often considered to have a dual ethnicity. In a day-to-day secular context, they are considered to be amaZulu, but when dealing with rainmaking and certain healing practices relating to rock art they are seen as San. Duma San identity is largely determined through the patrilineage, which is essentially a Nguni practice. Individuals descended from a mythical San founding hero, through the male line, regard themselves as essentially Muthwa (Bushman) (Frans Prins fieldnotes 2002 – 2008). However, the oral history is vague on the exact identity and context of this founding ancestor. Duma San descendants in the Kamberg Valley of the Central Drakensberg, maintain that this person was called Ngcezu (Francis 2007). It is said that he, and his followers, produced rock paintings but it is uncertain if they were still living as hunter-gatherers. Micheal Francis (2007) estimated that Ngcezu lived around 1840 at a time when Duma people were noted in historical records as living in the southern Drakensberg near Lotheni. According to local tradition, Ngcezu was invited by Dumisa, a charismatic leader of an essentially Zulu-speaking following (Vinnicombe 1976; Wright 1971), to settle amongst his people and to change their lifeway as a strategy to prevent retribution from colonial authorities for livestock raiding activities. Historical records are clear about San raiding livestock in the Drakensberg area during the 1830s until the early 1880s (Wright 1971). However, this practice incited colonial retribution, which would have led to severe social stress and new adaptations in terms of Mountain San responses to an ever-expanding colonial frontier (Prins 2009). According to local traditions, Ngcezu's descendants adopted the name Duma from their Nguni patron as a clever ploy to hide from the colonial settlers "who were constantly at the look-out for Bushmen" (Richard Duma: pers com 2003).

The adoption of a Nguni clan name and the ease at which these San apparently changed to a Zulu lifeway, suggest that this assimilation process probably started much earlier. Duma San elders interviewed by Prins in the greater Lotheni area (F E Prins fieldnotes 2005), in fact, maintain that their origins started some time earlier, when local San groups were invited to live with the Mpondomise, another Nguni group, who at the time lived at the coast of northern Transkei. It has been estimated that the Mpondomise, a group who had a long and intimate relationship with the

San (Jolly 1996; Wright 1971), lived at the coast during the 17th century, if not before (Derricourt 1974). Mpondomise traditions relate to a long period of cohabitation and intermarriage with San people, going back to at least the 17th century (Challis 2008; Jolly 1996).

The existing genetic composition, of the increasingly admixed San, could have been further affected by non-African populations who arrived and traded along the east coast of Africa. The 16th and 17th centuries saw various Portuguese shipwrecks along the coastline of the current KwaZulu-Natal and the Eastern Cape provinces. Many of the survivors of these shipwrecks were lascars (i.e., Muslim slaves of Indian origin) (Crampton 2004). According to Duma elders, some of these people of Indian origin did not follow their Portuguese masters back to Europe. Rather, they opted to stay on the southeastern coast of Africa, where they eventually intermarried with local inhabitants. It appears that San admixture at the coast intensified even more during this period, and Duma informants maintain that some Indian people intermarried with the local San, assimilating with them. Around the middle of the 18th century, the Mpondomise people left the coastal areas of the current Eastern Cape, and settled for a while along the middle reaches of the Mzimvubu River, eventually establishing themselves in the foothills of the Eastern Cape Section of the Drakensberg near Elliot (Derricourt 1974). It appears that they may have been accompanied by their San relatives and friends, who at this stage would have experienced marked acculturation.

By the beginning of the 19th century, some of the San appear to have left the Mpondomise, and settled to the north of them in the foothills of the southern KwaZulu-Natal Drakensberg. At this point, they were probably called the amaThola, an appellation they earned sometime earlier. According to Challis (2008), the name amaThola was most probably derived from the amaTholo – a Nguni-speaking agro-pastoralist people, who lived along the foothills of the southern KwaZulu-Natal Drakensberg in the early 1800s (Challis 2008). Challis found no evidence for the name amaThola in historical records in KwaZulu-Natal that predated 1835 (ibid). However, in 1803 Van Reenen Reported that there were 'yellow-skinned', but 'long-haired' people called "Matola" north of the Thembu in the present day Transkei (Derricourt 1974). These could very well have been Challis's amaThola who at that stage frequented the area between the Kei and Mzimvubu Rivers. The reference to the long hair may be suggestive of earlier intermarriage and admixture between San and Indian slaves, although this supposition needs to be assessed more

thoroughly. Nevertheless, the Indian ancestral contribution to the present Duma San is widely acknowledged. Locally, some of the Duma San are called the Sulumani. Duma informants are quick to point out their distinct long hair, aquiline features, and the fact that they still practice a form of Kosher (Albert Duma pers com 2011) (F Prins fieldnotes 2009-11).

According to Duma elders interviewed by F. Prins (fieldnotes 2009), the appellation amaThola was already used by their San forebears during the time of Shaka Zulu, when they were living in the southern KwaZulu-Natal Drakensberg. However, it changed first to amaTholweni then to Dumeni and finally to Duma, as more and more of them settled amongst Zulu-speaking agro-pastoralists becoming more sedentary. Apart from the Duma San of possible Indian descent (i.e., the Sulumani), they further self-identify as those of European or Coloured descent (i.e., amaColoured Duma), and those closest to their original San forebears called (Abathwa Duma). Informants maintain that early European or Coloured traders and adventurers often intermarried with their forebears, a fact also supported by historical documentation relating to Mountain San relationships with other groups in the greater ‘No man’s Land’ region during the 19th century (Challis 2008). It is further stated that the least admixed Duma San are those who still have the physical attributes of their San ancestors. It was the Abathwa San who usually took the lead in ritual matters, including initiating the Maqoma – a healing dance intimately associated with rock art production. They were also the rainmakers and the war doctors, and were often summoned by the Zulu King to strengthen his army (F Prins fieldnotes 2002 –11). Despite their diverse origins and divisions within the collective, all Duma sub-groups self-identify as San. Today, this highly admixed group is hardly distinguishable from their Zulu neighbors.

Ongoing visitations to rock art and other ‘sacred’ sites on the Drakensberg landscape is often expressed through the “invention of tradition”, where perceived San notions are reinterpreted by Zulu idiom. Perhaps the best known and researched example is the so-called Eland Ceremony where a typical Zulu concept, relating to ancestor veneration and pollution, have been applied to a major rock art site, namely Game Pass Shelter, in order to engage with the San ancestors in the spirit world (Duval 2014; Francis 2007; Ndlovu 2009; Prins 2009). Although the content and structure of the Eland Ceremony is essentially Zulu in nature, it nevertheless acts as an effective rite in maintaining a notion of ‘San-ness’ amongst present generation Duma. This is complimented

by Duma praise songs, again essentially a Nguni practice, as well as oral histories that refer to the ancestors of the present Duma San as Abathwa (San/Bushmen), in close association with certain wild animals and mountain caves. Today, most Duma San live at Lotheni in the foothills of the southern KwaZulu-Natal Drakensberg. However, smaller groups are scattered throughout the southern, central and northern Drakensberg.

Supplementary Note 4 (drafted by Carina Schlebusch)

The Karretjie People

’Karretjie’ is the Afrikaans word for a small vehicle, alluding to a mobile lifestyle based on the use of donkey carts. Throughout the great Karoo region of South Africa, there are small bands of people living this mobile lifestyle, but due to recent socio-economic changes, this lifeway is quickly disappearing. The Karretjie People phenotypically resemble Khoe-San people. Oral and archaeological records also suggest Khoe-San ancestry, but the group completely lost their original linguistic and socio-cultural affinities. They sometimes self-identify as Coloured and speak Afrikaans. Most of the Karretjie People are sheep shearers and fencers. Typically, they have a home base or as they call it *uitspan* or ‘outspan’ where they keep their cart in between jobs. These outspans are usually on a neutral piece of land such as the section of land between a road and a farm fence. They would stay in this space until their shearing or fencing skills were required by a farmer. When this happened, they would pack their donkey cart, and the whole family and living unit would move to the farm until the work was completed, after which they would move back to the same outspan (De Jongh 2002, 2004).

Genetic studies indicate a predominant San ancestry for the Karretjie People, with small amounts of Bantu-speaker and European admixture (coming mostly from the male side, following Y-chromosome studies) (Schlebusch et al. 2011; Schlebusch et al. 2012). The San ancestry is a southern San component – related to, e.g., the ǂKhomani. East African admixture is minimal (compared to the substantial component of East African ancestry in the KhoeKhoe group – e.g., the Nama (Breton et al. 2014; Schlebusch et al. 2012)), and therefore the ancestry of the Karretjie People is inferred to be mostly from southern San, rather than from Khoekhoe ancestors. The southern San groups living in these regions until the late 1800s were speakers of the southern San |Xam language.

The |Xam inhabited a region known as the great Karoo, an arid scrubland with dispersed hills stretching over 400 000 sq/km of the Northern, Eastern and Western Cape provinces of South Africa. This area was inhabited by both San and Khoekhoe groups up until the late 1800’s. The San group was the |Xam and the pastoralist Khoekhoe group was part of the Korana (!Ora) group (Barnard 1992). The |Xam had subgroups consisting of the Ss’wa ka (aka Plain Bushmen), |Nussa

(aka Grass Bushmen), !Kaoken ss’o (aka Mountain Bushmen and Brinkkop Bushmen), who all spoke the |Xam language with minor differences in dialect (Traill 1996). Much knowledge about the |Xam language was gained through the work of Wilhelm Bleek, a 17th century linguist (http://lloydbleekcollection.cs.uct.ac.za).

There are probably many reasons for the apparent disappearance of the |Xam. The principal factor probably is the advance of European hunters and farmers from the south in the 18th century, who then occupied all the hunting ground previously used by the |Xam. The occupation of their resources was not the only reason for the disappearance of the |Xam, they were actually hunted by colonists and bounties were placed on their heads (Penn 1996). Males that were not killed by hunters fled into the hilltops or were sent off to prison. Females and children were relocated to farms where they served as farmhands, the (aka tame Bushmen) (De Jongh 2002, 2004). Khoekhoe herders living in the area were also in competition with colonists for grazing land, but they claimed right to certain areas and owned cattle to trade with the Europeans. They therefore generally received more respect from colonists than San hunter-gatherers (Barnard 1992; Penn 1996; Traill 1996).

The descendants of some of the relocated |Xam females and children still live on European owned Karoo farms where they became admixed with the local Xhosa population (Bantu-speaking farmers). Amongst the older generation of European farmers, there are still a few who recognize the presence of labourers with San decent. Such persons were known to possess *wanderlust*, and refused to settle in one place. These people became the Karretjie People, with their donkey carts as mobile units, perpetually on the move to do different periodic jobs (De Jongh 2002, 2004).

Supplementary Note 5 (drafted by Carina Schlebusch)

The ǂKhomani

Several San groups lived in the far northern parts of the northern Cape (north of Upington), the southern part of Botswana and the southern parts of Namibia. Roughly where the Kgalagadi Tranfrontier Park is located today. They all spoke branches and dialects of the Tuu family (southern Khoisan). In 1980 there were only few individuals left in the region who remembered their traditional hunter-gatherer lifeway. They self-identified as San, but mostly spoke Nama (only few older individuals could speak the N||ng language). Ethnographic information indicates that in the past, the southern Kalahari San lived in small, scattered groups in the summer and aggregated in the area of the Nossob River (southern Botswana) during the winter. During such aggregation phases, they traded goods (such as ostrich eggshell beads and animal skins) with Bantu-speaking Tswana groups (Barnard 1992; Steyn 1984).

In the Northern Cape of South Africa (southern Kalahari), a group of descendants of these scattered southern Kalahari San groups now drew together under the term ǂKhomani San. They have recently won a land claim and have organized themselves into a community governed by a council. As mentioned above, only a few elderly individuals still spoke a San language, the N||ng language. The term ǂKhomani was not known to the N||ng speakers, it was introduced to San descendants of the Northern Cape by representatives of the South African San Institute (SASI). Other than N||ng, the only other extant Tuu languages are spoken in southern Botswana (Crawhall 2003; Sands et al. 2007).

Supplementary Note 6 (drafted by Carina Schlebusch)

Mitochondrial DNA and Y-chromosome haplogroups of the Lake Chrissie and Duma San

The combined Southern San and Bantu-speaker ancestry of the Lake Chrissie San was also apparent in the affinities of their mitochondrial DNA (mtDNA) and Y-chromosome haplogroups (see previously published results in (Schlebusch et al. 2013) and (Naidoo et al. 2010)). Two of the Chrissie San individuals carry L0d mtDNA haplogroups, L0d2a and L0d1b. The L0d haplogroup is the major mtDNA haplogroup in Khoe-San groups (Barbieri et al. 2014; Schlebusch et al. 2013). L0d2a and L0d1b, specifically, are the two most frequent L0d sub-haplogroups in the southern Khoe-San and also the two most common haplogroups in the Khoe-San descendent groups from South Africa (Schlebusch et al. 2013). The presence of L0d2a and L0d1b in the Chrissie San is therefore not unexpected. The remaining Chrissie San individual carry an L0a1b haplogroup. L0a haplogroups are found across central and eastern Africa and L0a occurs at its highest frequency in the Bantu-speakers of Mozambique (Salas et al. 2002). Since L0a occurs only at low frequencies in west Africa, it was likely incorporated into Bantu-speakers when they arrived in east Africa and admixed with local pre-Bantu-speaker east African groups. The presence of an L0a haplogroup in a Lake Chrissie San individual is thus also not unexpected (given the proximity of Lake Chrissie to Mozambique).

The Bantu-speaking associated Y-chromosome haplogroup E1b1a1 (previously E-M2) was present in two Lake Chrissie San individuals and the remaining individual belonged to Y-chromosome haplogroup B2a (previously B-M150). Since E1b1a1 and B2a are both very common haplogroups found in Bantu-speakers (de Filippo et al. 2011; Naidoo et al. 2010), Y-chromosomes suggest paternal line contributions from Bantu-speakers, in the Lake Chrissie San.

The Duma San had mtDNA haplogroups associated with both Bantu-speakers (L3d3, L3e1, L2a1f) and Khoe-San (L0d1b, L0d2a). This indicates both Bantu-speaker and Khoe-San contributions for the Duma San maternal line, as is correspondingly observed in South African Bantu-speakers (L0d is found in moderate frequencies (20-40%) in South African Bantu-speakers (Schlebusch et al. 2013)). The two Duma San males had Bantu-speaking Y-chromosome haplogroups (E1b1a1 and B2a).

SUPPLEMENTARY TABLES

Table S1 Internal classification of Southern African Khoisan linguistic group (Güldemann 2014)

**Lineages and branches**	**Languages and dialects**	**Remarks**
**Ju-ǂHõan (new name K'xa)**		
ǂ'Amkoe	Single language complex: ǂHõan, etc.	Newly affiliated to Ju
Ju (= Northern Khoisan)	Single language complex	
North	Angolan !Xuun varieties	
North-central	Ekoka !Xuun, Okongo !Xuun, etc.	
Central	Grootfontein !Xuun, etc	
Southeast	Ju|'hoan, ǂKx'au||'e	
**Khoe-Kwadi**		Possibly related to Sandawe
Kwadi	Single language	Newly affiliated to Khoe
Khoe (= Central Khoisan)		
Khoekhoe		
Cape Khoe	Language complex	
!Ora-Xiri	Language complex	
Eini	Language complex	
Nama-Damara	Language complex	
Hai||om	Single language	
ǂAakhoe		
Kalahari Khoe		
East		
Shua	Cara, Deti, |Xaise, Danisi, Ts'ixa, etc.	
Tshwa	Kua, Cua, Tsua, etc.	
West		
Kxoe	Khwe, ||Ani, Buga, G|anda, etc.	
G||ana	G||ana, G|ui, ǂHaba, etc.	
Naro	Naro, etc.	
**Tuu** (= Southern Khoisan)		
Taa-Lower Nossob		
Taa	Single language complex	
West	N|u||'en, West !Xoon	
East	'N|oha, N|amani, East !Xoon, Kakia, etc.	
Lower Nossob	|'Auni, |Haasi	
!Ui	N||ng: Langeberg, N|uu (= ǂKhomani or N|huki), etc.	
	Danster	
	Vaal-Orange	
	||Xegwi	
	!Ga'ne	
	|Xam: Strandberg, Katkop, Achterveld, etc	

Bold – Independent lineage;

Underlined – Earlier classification unit

Table S2- Sample and Comparative data description

Population	Source*	n	Place of origin	Linguistic grouping broad	Linguistic grouping narrow	Subsistence**
ǁXegwi from Lake Chrissie (CHR)	This study	3	South Africa	Khoisan (historically)	Tuu	Hunter-gatherer
Duma San (KZU)	This study	5	South Africa	Khoisan (based on oral traditions)	Tuu (based on oral traditions)	Hunter-gatherer (based on oral traditions)
Karretjie People	Schlebusch et al 2012	12	South Africa	Khoisan (historically)	Tuu	Hunter-gatherer
ǂKhomani	Schlebusch et al 2012	10	South Africa	Khoisan	Tuu	Hunter-gatherer
Coloured Askham	Schlebusch et al 2012	7	South Africa	Khoisan	Tuu+KhoeKhoe	Mixed
Nama	Schlebusch et al 2012	7	Namibia	Khoisan	KhoeKhoe	Pastoralist
ǀGui+ǁGana	Schlebusch et al 2012	7	Botswana	Khoisan	Kalahari-Khoe	Hunter-gatherer
Juǀ'hoansi	Schlebusch et al 2012	17	Namibia	Khoisan	Ju	Hunter-gatherer
!Xun	Schlebusch et al 2012	13	Angola	Khoisan	Ju	Hunter-gatherer
Khwe	Schlebusch et al 2012	17	Angola	Khoisan	Kalahari-Khoe	Hunter-gatherer
South Eastern Bantu	Schlebusch et al 2012	19	South Africa	Niger-Kordofanian	Southern Bantu	Agro-pastoralist
South Western Bantu (Herero)	Schlebusch et al 2012	8	Namibia	Niger-Kordofanian	Southwest Bantu	Pastoralist
Yoruba (YRI)	1000 genomes genotype data	20	Nigeria	Niger-Kordofanian	Atlantic-Congo	Agriculturist
Maasai (MKK)	1000 genomes genotype data	20	Kenya	Nilo-Saharan	Nilotic	Pastoralist
Luhya (LWK)	1000 genomes genotype data	20	Kenya	Niger-Kordofanian	Northeast Bantu	Agro-pastoralist
Northwestern European (CEU)	1000 genomes genotype data	20	Northwest Europe	Indo-European	Mixed	Agro-pastoralist
Tuscan (TSI)	1000 genomes genotype data	20	Italy	Indo-European	Italic	Agro-pastoralist
Japanese (JPT)	1000 genomes genotype data	20	Japan	Japonic	Japonic	Agriculturist
Amhara	Pagani et al 2012	20	Ethiopia	Afro-Asiatic	Semetic	Agro-pastoralist
Ari-Blacksmith	Pagani et al 2012	11	Ethiopia	Afro-Asiatic	Omotic	Agro-pastoralist
Gumuz	Pagani et al 2012	18	Ethiopia	Nilo-Saharan	Nilotic	Agriculturist
Oromo	Pagani et al 2012	20	Ethiopia	Afro-Asiatic	Cushitic	Agro-pastoralist
Somali	Pagani et al 2012	20	Somalia	Afro-Asiatic	Cushitic	Agro-pastoralist
Sudanese	Pagani et al 2012	20	South Sudan	Mixed (unknown)	Unknown	Mixed

* Comparative data was obtained from published sources (Auton et al. 2015; Pickrell et al. 2012; Schlebusch et al. 2012)

** Most subsistence practice classifications are based on historical information

Table S3a – Formal f3 tests of admixture

Target	Source1	Source2	f_3	std.err	Z
Chrissie San	YRI (West African)	Ju|'hoansi (San)	-0.00463	0.001009	-4.594
Chrissie San	MKK (East African)	Ju|'hoansi (San)	0.003395	0.001044	3.253
Chrissie San	AMHARA (East African)	Ju|'hoansi (San)	0.003612	0.001088	3.321
Chrissie San	CEU (European)	Ju|'hoansi (San)	0.006091	0.001128	5.399
Duma San	YRI (West African)	Ju|'hoansi (San)	-0.01033	0.000473	-21.835
Duma San	MKK (East African)	Ju|'hoansi (San)	-0.00123	0.000524	-2.34
Duma San	AMHARA (East African)	Ju|'hoansi (San)	-0.001555	0.000643	-2.420
Duma San	CEU (European)	Ju|'hoansi (San)	-0.00058	0.000692	-0.842
SE-Bantu-sp	YRI (West African)	Ju|'hoansi (San)	-0.009353	0.000225	-41.566
SE-Bantu-sp	MKK (East African)	Ju|'hoansi (San)	-0.001435	0.00027	-5.315
SE-Bantu-sp	AMHARA (East African)	Ju|'hoansi (San)	-0.004151	0.000356	-11.664
SE-Bantu-sp	CEU (European)	Ju|'hoansi (San)	0.000311	0.000422	0.735

Table S3b - f3 tests of SE-Bantu-speakers down sampled to n=5 (means of 100 independent down samplings)

Target	Source1	Source2	Mean f_3	S.dev f_3	Mean Z	S.dev Z
SE-Bantu-sp	YRI (West African)	Ju|'hoansi (San)	-0.00976431	0.001325063	-20.7522	3.287523
SE-Bantu-sp	MKK (East African)	Ju|'hoansi (San)	-0.00199174	0.001381181	-3.81145	2.683771
SE-Bantu-sp	AMHARA (East African)	Ju|'hoansi (San)	-0.0009572	0.001371626	-1.6829	2.362224
SE-Bantu-sp	CEU (European)	Ju|'hoansi (San)	-0.00081485	0.001300011	-1.19633	1.870384

Table S4 - Admixture dates as determined by admixture linkage disequilibrium (LD) decay patterns (Patterson et al. 2012)

Admixed	Source1	Source2	Mean Generations	Mean years (25 y/gen)	Residual-SE	Sig Code[1]
**Amdixture of west Africans into various Khoe-San groups**						
Duma San	YRI (West African)	Ju|'hoansi (San)	33.2	830	0.002885	333
Karretjie People	YRI (West African)	Ju|'hoansi (San)	8.2	205	0.002497	333
ǂKhomani	YRI (West African)	Ju|'hoansi (San)	14.6	365	0.002461	333
|Gui+||Gana	YRI (West African)	Ju|'hoansi (San)	22.1	553	0.002601	333
!Xun	YRI (West African)	Ju|'hoansi (San)	27.2	680	0.002746	333
**Amdixture of Khoe-San into Bantu-speakers (with west African origin)**						
SE-Bantu-sp	YRI (West African)	Ju|'hoansi (San)	31.2	780	0.001461	333
SW-Bantu-sp	YRI (West African)	Ju|'hoansi (San)	40.7	1018	0.001447	333

[1] SigCode - Significance code - the level of significance of the three terms "A", "C" and "m" of the exponential fit given by the formula "wcorr ~ (C + A * exp(-m * dist/100))". Where "3" is pval <= 0.001; "2" is pval <= 0.01; "1" is pval <= 0.05 and "0" is pval > 0.05. Thus "333" would mean that all three terms in the exponential fit formula had significant p-values of <0.001 while with "000" none of the terms were significant.

SUPPLEMENTARY FIGURES

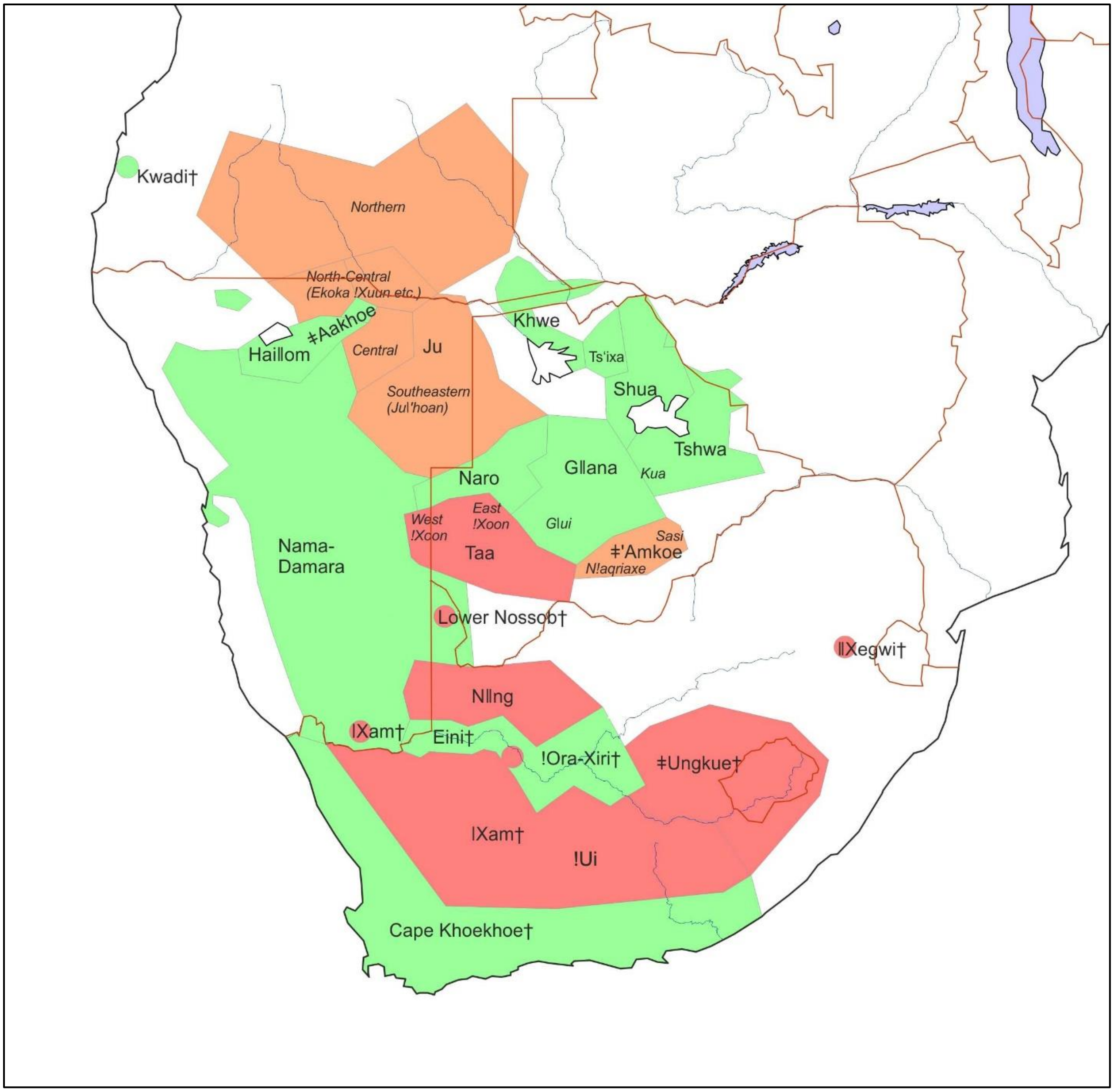

Figure S1 – Map of Khoisan linguistic phylum sub-group distribution (See Table S1). Orange – Ju (Northern Khoisan), Green – Khoe (Central Khoisan), Red – Tuu (Southern Khoisan).

† Extinct language

Map with permission from Tom Güldemann (Güldemann 2015)

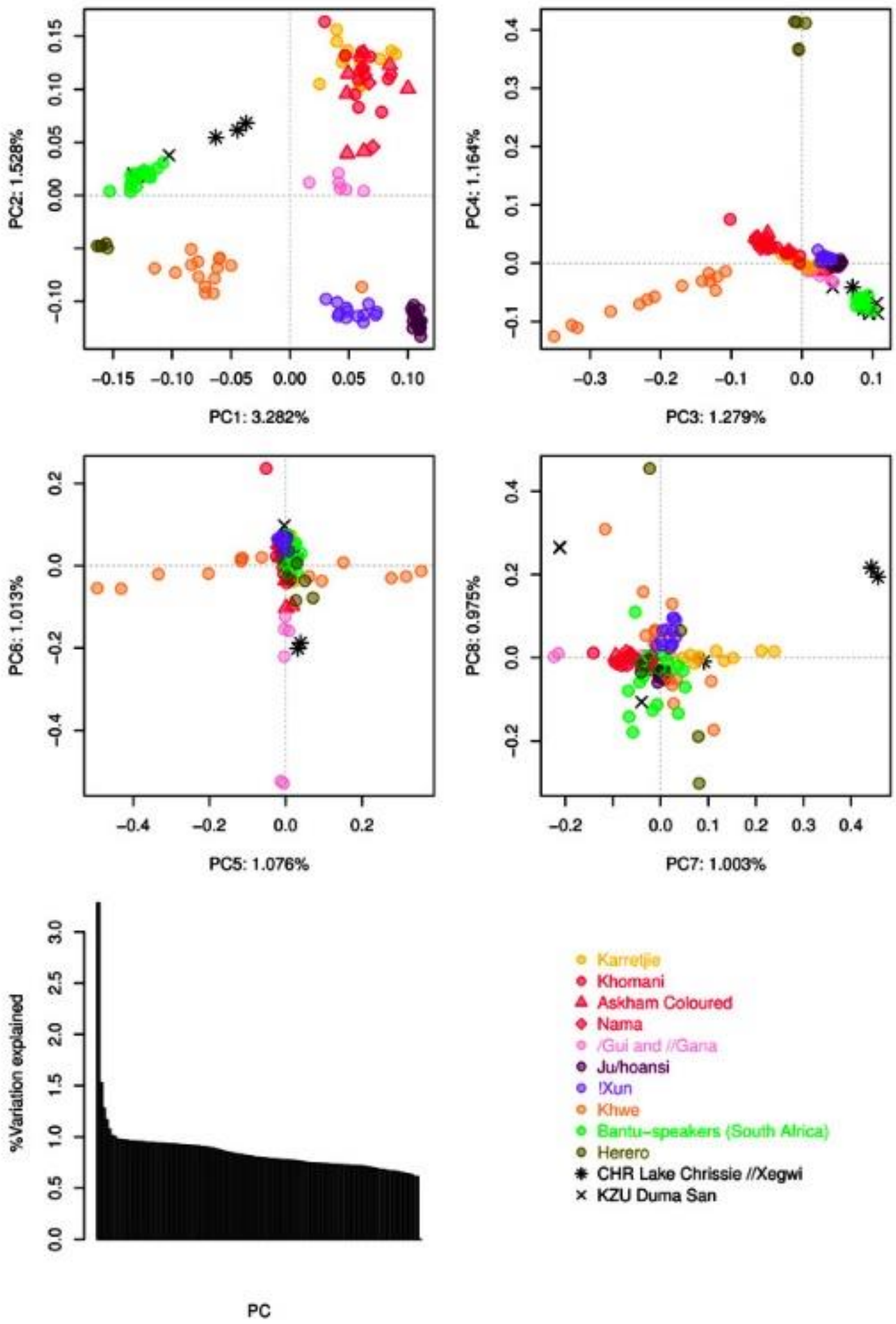

Figure S2 – PCA on the southern African dataset

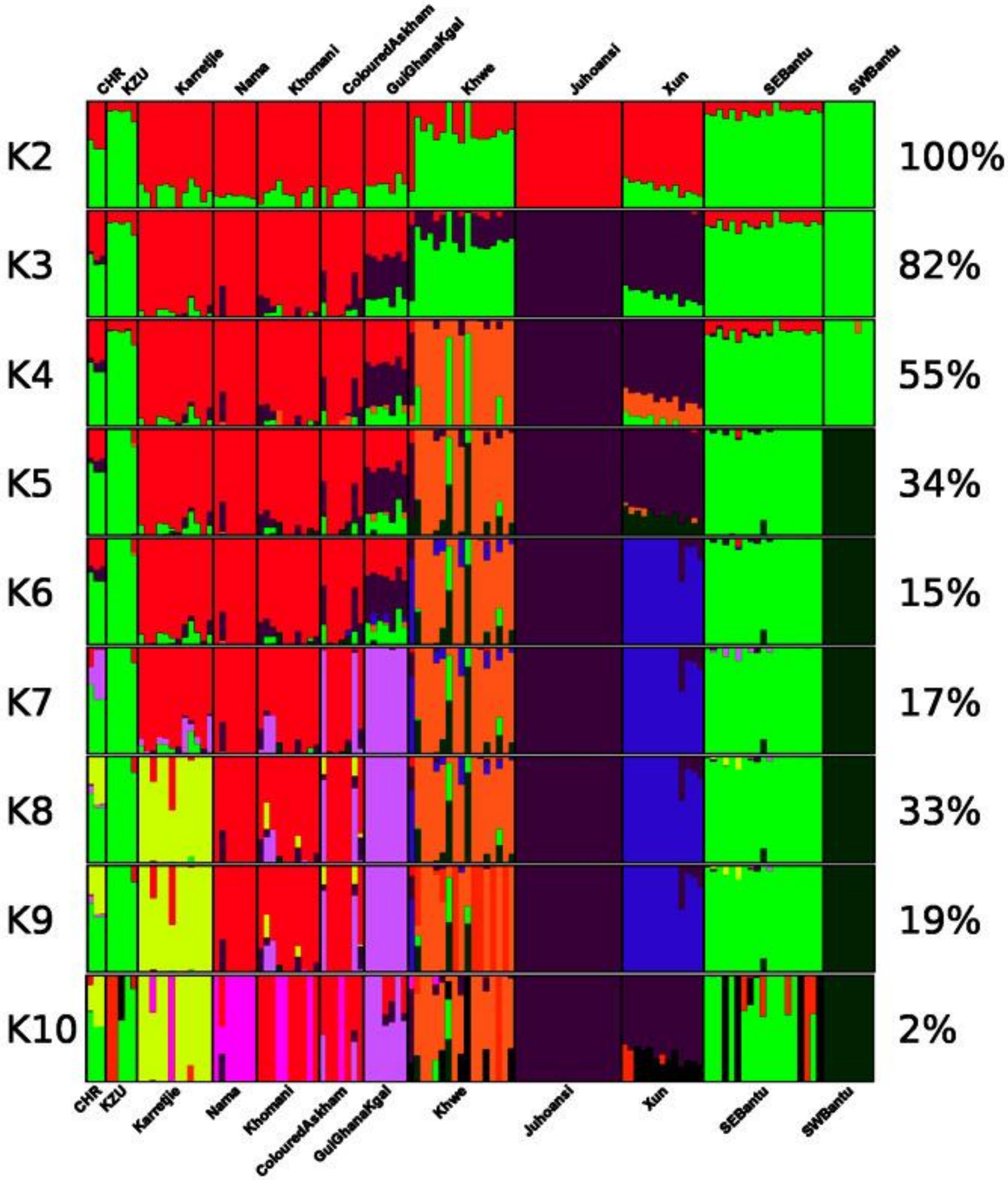

Figure S3 – Admixture analysis on the southern African dataset. See table S2 for population codes and descriptions.

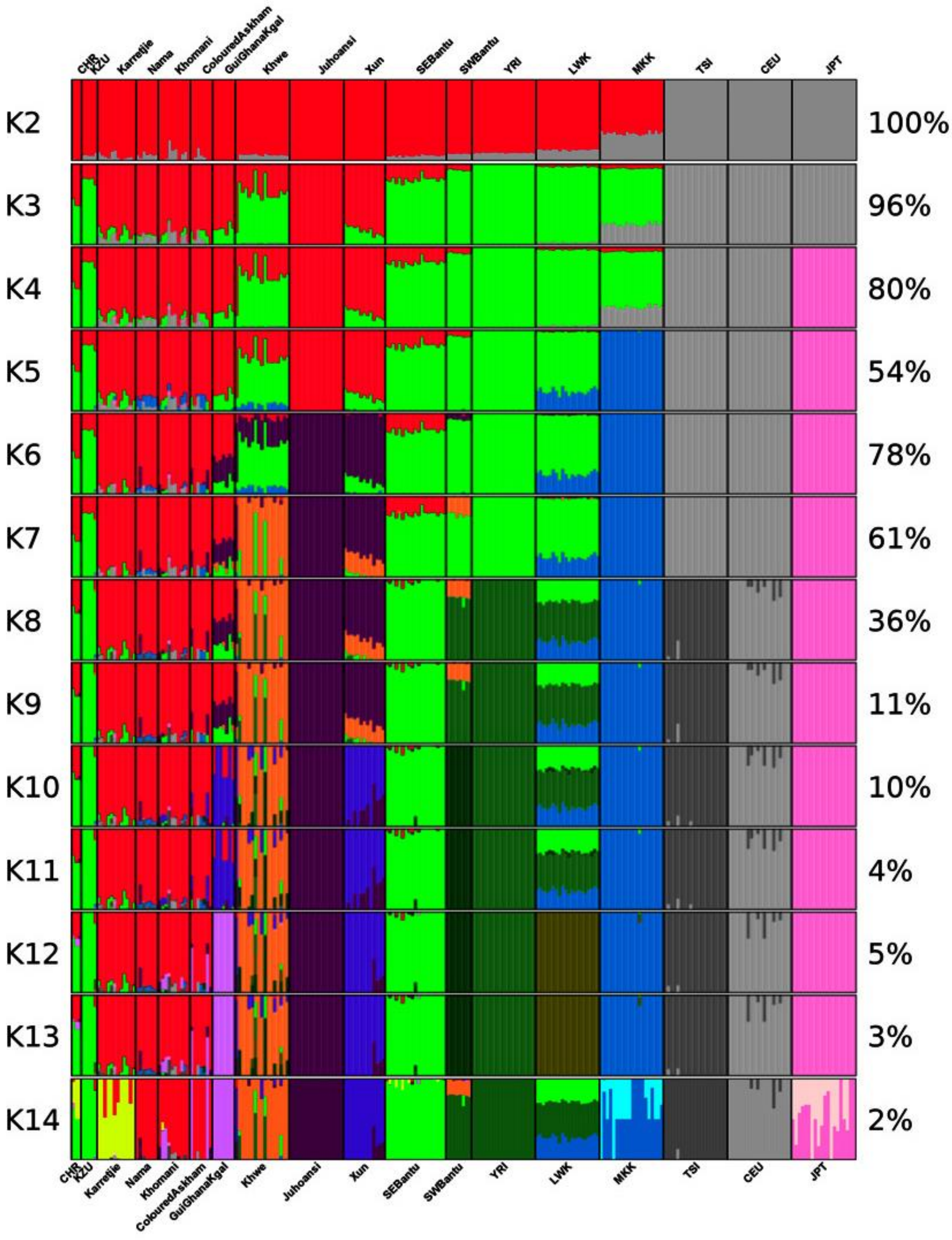

Figure S4 – Admixture analysis on the KGP extended dataset. See table S2 for population codes and descriptions.

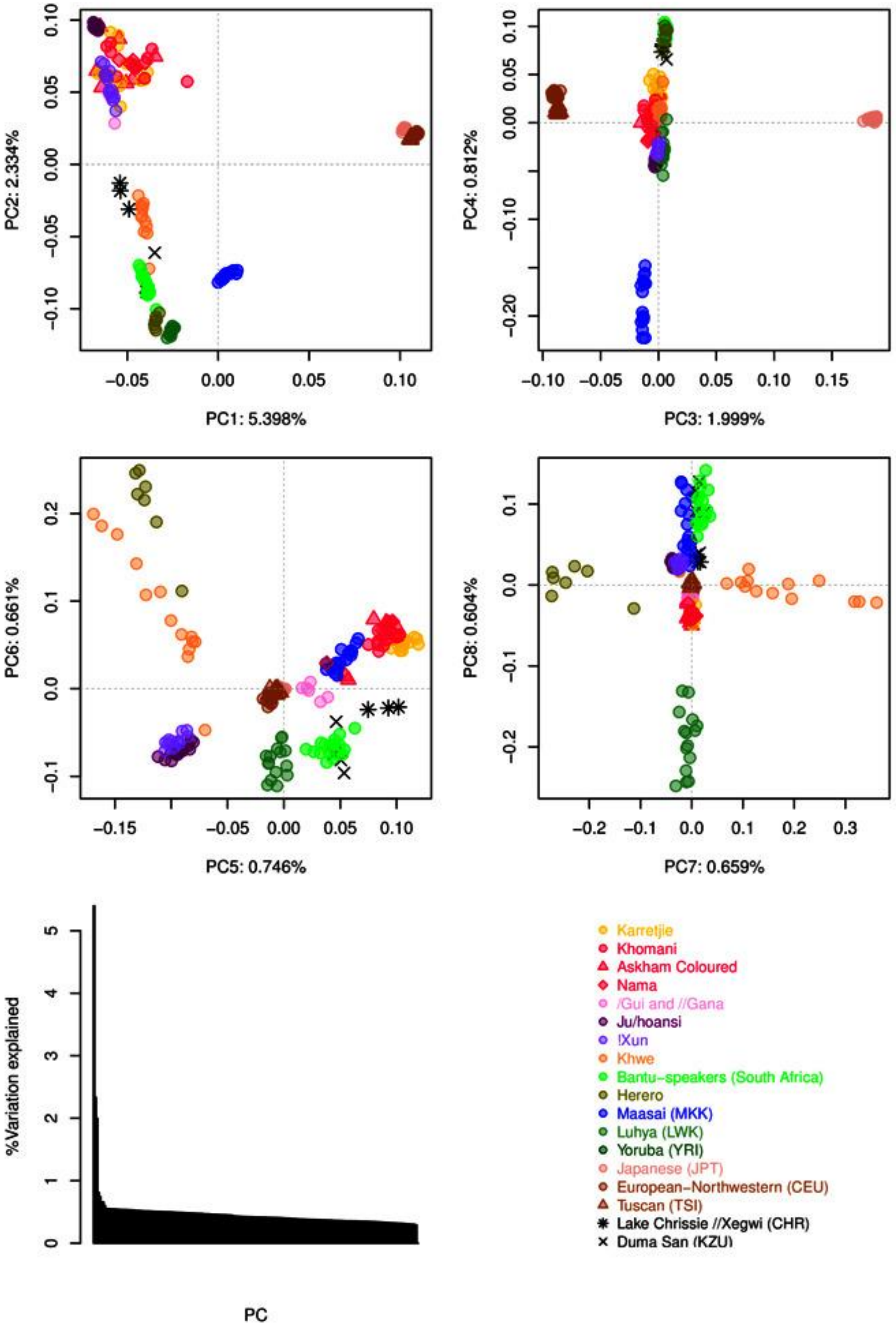

Figure S5 – PCA on the KGP extended dataset

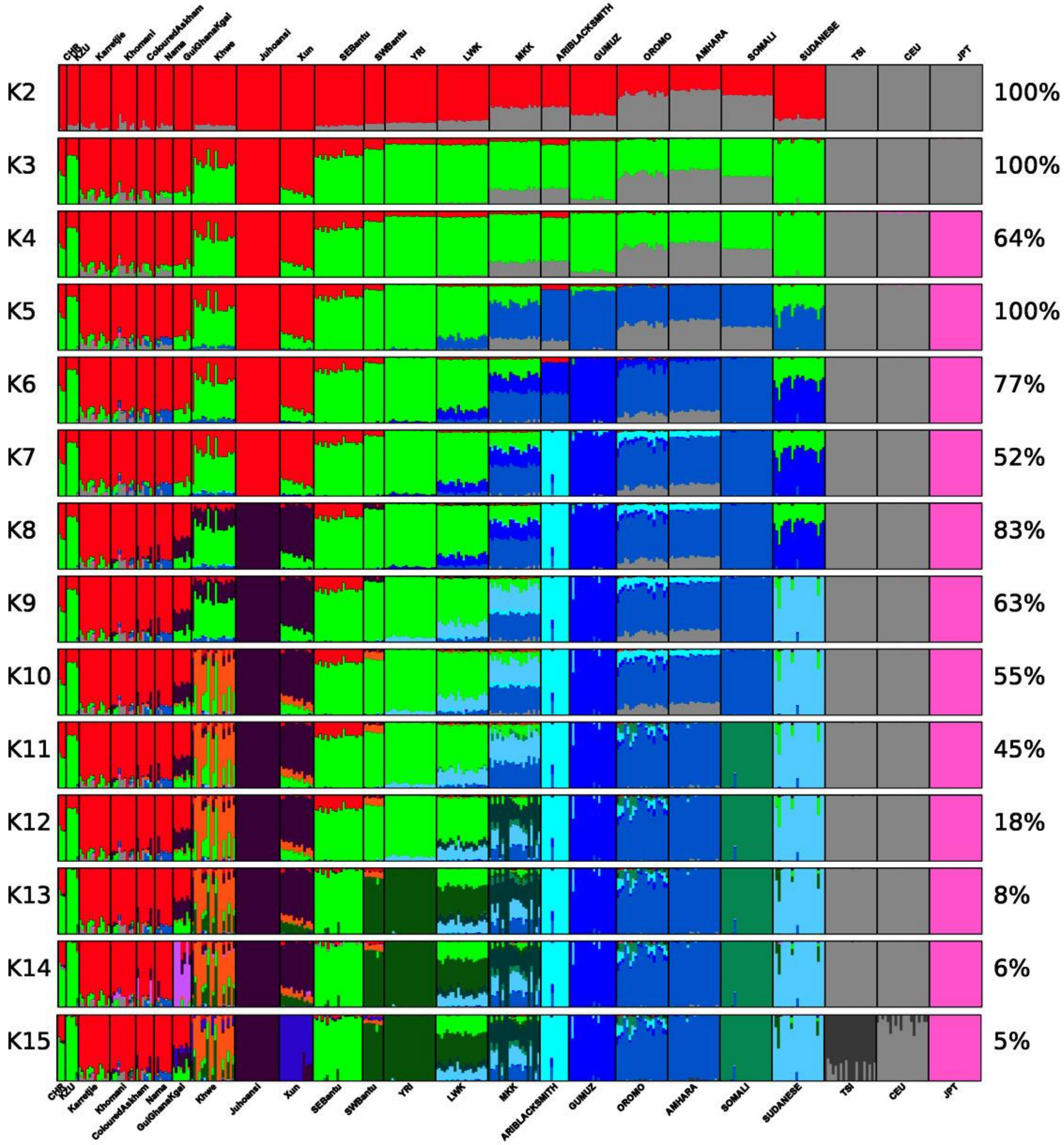

Figure S6 – Admixture analysis on the East African extended dataset. See table S2 for population codes and descriptions.

SUPPLEMENT REFERENCES

Auton A, Brooks LD, Durbin RM, Garrison EP, Kang HM, Korbel JO, Marchini JL, McCarthy S, McVean GA, Abecasis GR (2015) A global reference for human genetic variation. Nature 526: 68-74

Barbieri C, Guldemann T, Naumann C, Gerlach L, Berthold F, Nakagawa H, Mpoloka SW, Stoneking M, Pakendorf B (2014) Unraveling the complex maternal history of Southern African Khoisan populations. Am J Phys Anthropol 153: 435-48

Barnard A (1992) Hunters and herders of southern Africa - A comparitive ethnography of the Khoisan peoples. Cambridge University Press, Cambridge

Breton G, Schlebusch CM, Lombard M, Sjodin P, Soodyall H, Jakobsson M (2014) Lactase persistence alleles reveal partial East african ancestry of southern african Khoe pastoralists. Curr Biol 24: 852-8

Challis W (2008) The Impact of the Horse on the Amatola "Bushmen": New Identity in the Maloti-Drakensberg Mountains of Southern Africa. Unpublished PhD thesis, University of Oxford

Crampton H (2004) The Sunburnt Queen. Jacana Press, Johannesburg

Crawhall N The rediscovery of N|u and the ‡Khomani Land Claim Process, South Africa Maintaining the Links: Language Identity and the Land: Proceedings of the Seventh Foundation for Endangered Languages Conference, Broome, Western Australia 2003. Bristol: Foundation for Endangered Languages, pp 13-19

Crawhall N (2006) Languages, genetics and archaeology: problems and the possibilties in Africa. In: Soodyall H (ed) The prehistory of Africa. Jonathan Ball Publishers, Johannesburg & Cape Town, pp 109-124

de Filippo C, Barbieri C, Whitten M, Mpoloka SW, Gunnarsdottir ED, Bostoen K, Nyambe T, Beyer K, Schreiber H, de Knijff P, Luiselli D, Stoneking M, Pakendorf B (2011) Y-chromosomal variation in sub-saharan Africa: insights into the history of Niger-Congo groups. Mol Biol Evol 28: 1255-69

De Jongh M (2002) No fixed abode: the poorest of the poor and elusive identities in rural South Africa. Journal of Southern African Studies 28: 441-460

De Jongh M (2004) Strangers in Their Own Land: Social Resources and Domestic Fluidity of the Peripatetic Karretjie People of the South African Karoo. In: Berland JC, Rao A (eds) Customary Strangers. New perspectives on peripatetic peoples in the Middle East, Africa and Asia. Praeger, London, pp 155-178

Derricourt R (1974) Settlement in the Transkei and Ciskei before the Mfecane. In: Saunders C, Derricourt R (eds) Beyond the Cape frontier. Longman, London

Duval M (2014) Enjeux patrimoniaux et identitaires autour des sites d'art rupestre sud-africains. Civilisations 61: 83-101

Filter H (1925) Die Buschmänner. Hermannsburger Missionsblatt 10: 186-189

Francis M (2007) Explorations in Ethnicity and Social Change among Zulu-speakinbg San descendants of the Drakensberg Mountains, KwaZulu-Natal. Unpublished PhD thesis, University of KwaZulu-Natal

Güldemann T Kx'a, Tuu, and Khoe foragers in the Kalahari Basin core. Paper presented at the International Symposium '"Speaking (of) Khoisan": a symposium reviewing southern African prehistory'. MPI for Evolutionary Anthropology Leipzig. 14-16 May 2015 2015

Güldemann T (2014) "Khoisan" linguistic classification today. In: Güldemann T, Fehn A-M (eds) Beyond ‘Khoisan’: historical relations in the Kalahari Basin. Current Issues in Linguistic Theory 330. John Benjamins, Amsterdam, pp 1-41

Jolly P (1996) Interaction between south-eastern San and southern Nguni and Sotho communities, c. 1400 to c. 1800. South African Historical Journal 35: 30-61

Mitchell P (1990) Preliminary report on the Later Stone Age sequence from Tloutle Rock Shelter, Western Lesotho. South African Archaeological Bulletin 12 24: 100

Naidoo T, Schlebusch CM, Makkan H, Patel P, Mahabeer R, Erasmus JC, Soodyall H (2010) Development of a single base extension method to resolve Y chromosome haplogroups in sub-Saharan African populations. Investig Genet 1: 6

Ndlovu N (2009) Access to Rock Art Sites: A Right or a Qualification. South African Archaeological Bulletin 64: 61-68

Patterson N, Moorjani P, Luo Y, Mallick S, Rohland N, Zhan Y, Genschoreck T, Webster T, Reich D (2012) Ancient admixture in human history. Genetics 192: 1065-93

Penn N (1996) "Fated to Perish": The Destruction of the Cape San. In: Skotnes P (ed) Miscast. Negotiating the Presence of the Bushmen. UCT Press, Cape Town, pp 81-91

Pickrell JK, Patterson N, Barbieri C, Berthold F, Gerlach L, Guldemann T, Kure B, Mpoloka SW, Nakagawa H, Naumann C, Lipson M, Loh PR, Lachance J, Mountain J, Bustamante CD, Berger B, Tishkoff SA, Henn BM, Stoneking M, Reich D, Pakendorf B (2012) The genetic prehistory of southern Africa. Nat Commun 3: 1143

Potgieter EF (1955) The disappearing Bushmen of Lake Chrissie: a preliminary survey. J.L. van Schaick, Pretoria

Prins FE (1999) A Glimpse into Bushman Presence in the Anglo-Boer War. Natalia 29: 51-59

Prins FE (2001) Rock Art and Motivation: The Evidence from Magageng. Pictogram 12: 14-18

Prins FE (2009) Secret San of the Drakensberg and their rock art legacy. Critical Arts 23 190-208

Salas A, Richards M, De la Fe T, Lareu MV, Sobrino B, Sanchez-Diz P, Macaulay V, Carracedo A (2002) The making of the African mtDNA landscape. Am J Hum Genet 71: 1082-111

Sanders T (2013) Lake Chrissie's Bushman Past. Highveld Printers, Ermelo

Sands BE, Miller AL, Brugman J (2007) The Lexicon in Language Attrition: The Case of N|uu. In: Payne DL, Peña J (eds) Selected Proceedings of the 37th Annual Conference on African Linguistics. Cascadilla Proceedings Project, Somerville, MA, pp 55-65

Schapera I (1930) The Khoisan Peoples of South Africa: Bushmen and Hottentots. George Routledge and Sons, London

Schlebusch CM, de Jongh M, Soodyall H (2011) Different contributions of ancient mitochondrial and Y-chromosomal lineages in 'Karretjie people' of the Great Karoo in South Africa. J Hum Genet 56: 623-30

Schlebusch CM, Lombard M, Soodyall H (2013) MtDNA control region variation affirms diversity and deep sub-structure in populations from Southern Africa. BMC Evol Biol 13: 56

Schlebusch CM, Skoglund P, Sjodin P, Gattepaille LM, Hernandez D, Jay F, Li S, De Jongh M, Singleton A, Blum MG, Soodyall H, Jakobsson M (2012) Genomic Variation in Seven Khoe-San Groups Reveals Adaptation and Complex African History. Science 338: 374-379

Schoonraad M, Schoonraad E (1972) Rotskuns van Oos Transvaal. Outlook 6: 8-11

Schultze L (1928) Zur Kenntnis des Korpers der Hottentotten und Buschmanner. Zoologische und Anthropologische Ergebnisse einer Forschungsreise im westlichen und zentralen Sudafrika, vol 5, pp 147-227

Steyn HP (1984) Southern Kalahari San Subsistence Ecology: A Reconstruction. The South African Archaeological Bulletin 39: 117-124

Traill A (1996)!Khwa-Ka Hhouiten Hhouiten - "The Rush of the Storm" : The linguistic death of /Xam. In: Skotnes P (ed) Miscast. Negotiating the Presence of the Bushmen. UCT Press, Cape Town, pp 171-183

Vinnicombe P (1976) People of the eland: rockpaintings of the Drakensberg Bushmen as a reflection of their life and thought. University of Natal Press, Pietermaritzburg

Wright JB (1971) Bushman raiders of the Drakersberg, 1840-1870. University of Natal Press, Pietermaritzburg
